# Protected to death: systematic exclusion of pregnant women from Ebola virus disease trials

**DOI:** 10.1186/s12978-017-0430-2

**Published:** 2017-12-14

**Authors:** Melba F. Gomes, Vânia de la Fuente-Núñez, Abha Saxena, Annette C. Kuesel

**Affiliations:** 1 World Health Organization, Geneva, Switzerland; 2 Department for Ageing and Life Course, World Health Organization, Geneva, Switzerland; 3 Department for Information Evidence and Research, World Health Organization, Geneva, Switzerland; 4 UNICEF/UNDP/World Bank/WHO Special Programme for Research and Training in Tropical Diseases, World Health Organization, Geneva, Switzerland

**Keywords:** Pregnancy, Exclusion-criteria, Ebola, Ethics, Risk-benefit, Epidemic, Trials, Research

## Abstract

**Background:**

For 30 years, women have sought equal opportunity to be included in trials so that drugs are equitably studied in women as well as men; regulatory guidelines have changed accordingly. Pregnant women, however, continue to be excluded from trials for non-obstetric conditions, though they have been included for trials of life-threatening diseases because prospects for maternal survival outweighed potential fetal risks. Ebola virus disease is a life-threatening infection without approved treatments or vaccines. Previous Ebola virus (EBOV) outbreak data showed 89–93% maternal and 100% fetal/neonatal mortality. Early in the 2013–2016 EBOV epidemic, an expert panel pointed to these high mortality rates and the need to prioritize and preferentially allocate unregistered interventions in favor of pregnant women (and children). Despite these recommendations and multiple ethics committee requests for their inclusion on grounds of justice, equity, and medical need, pregnant women were excluded from all drug and vaccine trials in the affected countries, either without justification or on grounds of potential fetal harm. An opportunity to offer pregnant women the same access to potentially life-saving interventions as others, and to obtain data to inform their future use, was lost. Once again, pregnant women were denied autonomy and their right to decide.

**Conclusion:**

We recommend that, without clear justification for exclusion, pregnant women are included in clinical trials for EBOV and other life-threatening conditions, with lay language on risks and benefits in information documents, so that pregnant women can make their own decision to participate. Their automatic exclusion from trials for other conditions should be questioned.

## Background

The 2013–2016 Ebola virus (EBOV) epidemic was estimated to have caused 28,616 confirmed, probable, and suspected cases and 11,310 deaths [1], but the true burden of EBOV may have been higher. The number of cases and deaths exceeded by more than two orders of magnitude those across all 29 previous outbreaks [1–3]. No approved treatments or vaccines were available and a large number of trials were initiated.

Inclusion of women in trials submitted for US registration improved after 1993, when new regulatory guidelines required that a representative sample of patients likely to receive a drug be included in clinical studies and data be analyzed to determine gender differences in response [4–8]. The gender guidelines were developed amidst growing concerns that the drug development process did not provide adequate information about the effects of drugs or biological products in women, particularly HIV treatments, and a general consensus that women should have autonomy to determine participation in clinical trials for themselves [5, 8, 9]. Women are now generally included in trials—provided they are not pregnant and commit, as necessary, to effective birth control [9]. Exclusion of pregnant women is still usual practice in trials that do not address obstetric conditions, largely due to concern about birth defects after specific drug exposure in utero and the view that high fetal risk without important medical benefits for the mother is not acceptable [4, 9, 10]. Exclusion, therefore, should not apply to women with life-threatening diseases, as illustrated by early HIV/AIDS drug trials which included pregnant women in the earliest phases—before completion of animal-reproduction studies—because any risk to the fetus was balanced by an overwhelming potential benefit (prolonging life) to the mother [11]. The absence of data on general medical conditions in pregnancy means that pregnant women continue to be treated for non-obstetric conditions with drugs which did not undergo rigorous scientific testing in pregnancy, and for which safe and effective therapeutic doses in pregnancy and maternal and fetal risks are largely unknown [8–10, 12, 13]. More pregnant women and their future offspring are therefore exposed to potential harms through off-label use of medications than would be the case with rigorous scientific testing of medications used during pregnancy [14, 15].

In this paper, we review the case fatality data for pregnant women and fetuses/neonates from previous outbreaks and the pregnancy-related eligibility criteria of the therapeutic and vaccine studies in EBOV-affected countries. In the absence of registered treatments or vaccines to control this lethal disease, the World Health Organization (WHO) coordinated and supported research to expedite identification of interventions that could control the outbreak and improve future control efforts. Furthermore, WHO supported many of these studies, which therefore required WHO Ethics Review Committee (WHO-ERC) approval [16]. We reflect on how WHO-ERC made decisions regarding the eligibility of pregnant women during the 2013–2016 outbreak and provide an overview of the case fatality data now available to inform research during future outbreaks.

### Data on maternal and pregnancy outcomes informing study protocols in the 2013–2016 EBOV epidemic

Table 1 summarizes published data from EBOV outbreaks on maternal and pregnancy outcomes, Section A for the previous epidemics, Section B for the 2013-6 epidemic. In the 1976 outbreak, the case fatality rate (CFR) in EBOV-infected pregnant women was 89% (73/82) [17]. Almost half of all EBOV-infected women were pregnant (46%: 82/177). The high risk in pregnancy was later attributed to the repeated use of needles for vitamin injections in routine antenatal care without sterilization between patients [17, 18]. In the 1995 outbreak, 15/105 (14%) EBOV-infected women were pregnant [17]. The CFR for EBOV-infected pregnant women was 93% (14/15) compared with 70% (28/40) for EBOV-infected non-pregnant women and an overall 77.5% CFR (245/316) [17]. The differences in CFR are not statistically significant.

**Table 1:** Published data on maternal and pregnancy outcomes after EBOV infection

Location (Time) [Reference]	Number of pregnant women	Pregnancy stage	Age	Maternal outcome	Pregnancy outcome
A. Data available from previous epidemics to inform design of studies in the 2013–2016 EVD epidemic					
Southern Sudan (1976) [69]	NS	NS	NS	NS	Occasional premature labour
Zaire, Yambuku (1976) [18]	82	NS	NS	Died: 73/82 (89%)	Spontaneous abortion:
					18/73 who died
					1/9 who survived
					Live births to women who died: 11 (0 surviving beyond 19 days)
Zaire, Kikwit (1995) [17]	15	1st: 4 (25%)	Mean: 32	Died: 14/15 (93%)	Spontaneous abortion:
		2nd: 6 (40%)	Range: 24–38		9/14 who died
		3rd: 5 (33%)			1/1 who survived
					Stillbirth at 32 weeks: 1
					Full term live delivery: 1 (newborn died 3 days later, mother died due to extreme genital bleeding)
Gulu, Uganda [70]	1	28 weeks	30	Discharged alive	Spontaneous abortion
DRC, Isiro (2012) [71]	1	7 months	29	Died one day after delivery	Premature delivery on day 6 of disease, newborn died at 8 days
B. Data emerging during the 2013–2016 EVD epidemic					
DRC, Equateur (July-Oct 2014) [47]	1	NS	NS	Died	Died in utero with mother
Liberia, Monrovia (after August 2014)^a^ [48]	1	Late stage	31	Died	Died in utero with mother
Liberia, Monrovia (Aug - Oct 2014)^a^ [53]	4	Late 2nd /3rd	NS	Died: 3 (75%)	Miscarriage shortly before maternal death: 3
					Fetus carried to term: 0
Guinea, Guéckédou (Feb/March 2014)^b^ [50]	1	NS	16	Died^g^	Spontaneous abortion
Guinea, Guéckédou (June 2014)^c^ [51]	2	7 months	20s	Survived	Stillbirths
Guinea, Guéckédou (Dec 2014/Jan 15)^c^ [52]	2	4 months; 5 months	40/22	Survived	Miscarriages
Guinea, Conakry (2015) [56]	1	35–36 weeks	25	Died day of delivery (treated with favipiravir outside, but as per JIKI trial procedures) [38]	Live girl, EBOV qRT-PCR positive, monitored emergency use of ZMapp on days 2, 5, 8; buffy coat transfusion from EVD survivor on day 11
Sierra Leone, Kailahun (2014–2015)^d^ [57]	1	36 weeks	34	Survived	Induced delivery after diagnosis of intrauterine fetal death
Sierra Leone, Kailahun, Kenema (May/June 2014) [55]	1	35^h^	NS	Died^h^	Miscarriage
Sierra Leone, Bo (2014/2015)^e^ [58]	1	7 months	20	Survived	Stillborn fetus
Guinea, Sierra Leone, Liberia MSF Ebola Treatment Centers (2014–2016) [56]	54^f^	2nd/3rd trimester	NS	NS	2nd trimester miscarriages: 35
					Neonatal death: 1 (after 2 days)
Guinea, Sierra Leone, Liberia MSF Ebola Treatment Centers (1 April 2014–15 April 2015)^f^ [54] [49]	77^f^ [54]	1st: 16		41/77 died:	Stillbirth: 30
				22 undelivered	Neonatal death: 1
		2nd: 26		18 delivered before death 36/77 survived [49] [54]	
		3rd: 28			
		Missing information:7			
Guinea & Sierra Leone (April 2015–2016)^i^ [43] [72]	>20	NS			2 spontaneous abortions

*DRC* Democratic Republic of the Congo, formerly Zaire, *NS* not specified

^a^ELWA 3 MSF Ebola Treatment Unit, ^b^MSF team, ^c^MSF Ebola Teatment Centre Guéckédou, ^d^MSF Ebola Treatment Unit Kailahun, ^e^MSF Ebola Management Center Bo, ^f^Data on 77 women from eight MSF Ebola Management Centers includes 12 women in the 2nd and 3rd trimester reported in publications [48] [50] [51] [52] [53] [57]

^g^Severine Caluwaerts, personal communication: this woman is incorrectly reported in the publication as having survived

^h^J.S. Schieffelin, personal communication; outcome not reported in the paper [55]

^i^A-M Henao-Restrepo and MSF, personal communication

In aggregate, any EBOV-infected pregnant woman had survived only after spontaneous miscarriage, elective abortion, stillbirth, or with a neonatal death (Table 1 SectionA). All EBOV-infected pregnant women developed vaginal and uterine bleeding and were at high risk for spontaneous abortion and pregnancy-related hemorrhage [19]. In the 1976 outbreak, the rate of spontaneous abortions was 23% (19/82). The remaining pregnancy outcomes were stillbirths or neonatal deaths—no neonate survived longer than 19 days [18]. In 1995, the spontaneous abortion rate was 67% (10/15), with three elective abortions, one premature stillborn, and one live-born, full-term neonate who died at three days [17]; one of the three elective abortions followed an incomplete spontaneous abortion and the woman survived [17]. Four EBOV-infected mother-baby pairs were traced after the 2000–2001 outbreak in Uganda: all mothers and babies had died [20, 21].

### Clinical trials of potential treatments and vaccines during the 2013–2016 epidemic in Guinea, Liberia, and Sierra Leone

At the time of this epidemic, there were no approved specific treatments or vaccines for Ebola virus disease (EVD). Clinical management consisted of supportive care, particularly fluid and electrolyte management, correction of coagulopathy, treatment of secondary infections, and management of other complications [19]. Treatments proposed had not undergone clinical trials in EBOV populations or at all [22–24]. Vaccines were in very early development with few having entered Phase I safety and immunogenicity trials [24–29].

Table 2 lists the trials conducted during the 2013–2016 epidemic in Liberia, Guinea, and Sierra Leone and their pregnancy-related eligibility criteria. All drug and vaccine trials excluded pregnant women. Two of three convalescent plasma studies, funded by the European Union, included pregnant women [30]. Pregnant women were granted access to new treatments only within ‘Monitored Emergency Use of Unregistered Interventions’ (MEURI) [31] protocols implemented by Médecins Sans Frontières (MSF) for MIL77 (three chimeric monoclonal antibodies targeting different epitopes on the surface of EBOV glycoprotein) and favipiravir [16, 31].

**Table 2:** Drug and vaccines trials proposed, initiated, or completed during the 2013–2016 Ebola virus disease epidemic in Guinea, Liberia, and Sierra Leone

Clinical trial registry ID	Study type (Phase)^a^	Intervention	Scientific title [References to publications]	Country	Status	WHO-ERC Review (WHO-supported)	Pregnant women excluded (test)^a,b^
ISRCTN17414946	Treatment (NS)	IFN ß-1a	A pilot study to evaluate the safety and efficacy of interferon beta-1a (IFN ß-1a) in the treatment of patients presenting with Ebola virus illness	Guinea	<10 patients enrolled, results pending [73]	No (No)	Yes (NS)
PACTR201411000939962	Treatment (NS)	Brincidofovir	Open-label, non-randomised single arm trial to investigate the efficacy of brincidofovir compared to historic controls for Ebola virus disease in an outbreak setting in West Africa (RAPIDE-BCV) [32]	Liberia	Completed	Yes (No)	Yes (Yes)
NCT02329054	Treatment (2)	Favipiravir	Efficacy of favipiravir in reducing mortality in individuals with Ebola Virus Disease in Guinea (JIKI) [38] [74]	Guinea	Completed	Yes (No)	Yes (Yes)
NCT02662855	Treatment (2)	Favipiravir	Efficacy of favipiravir against severe Ebola virus disease	Sierra Leone	Completed	No (No)	Yes (NS)
ChiCTR-OCN-15007272	Treatment (NS)	Favipiravir	Clinical and virological characteristics of Ebola Virus Disease patients treated with favipiravir (T-705) - Sierra Leone, 2014 [75]	Sierra Leone	Completed	No (No)	NS (NS)
PACTR201501000997429	Treatment (NS)	TKM-130803	Open-label, single arm trial to investigate the efficacy of TKM-130803 with a concurrent observational study of Ebola virus Disease in an outbreak setting in West Africa (RAPIDE TKM) [40] [76]	Sierra Leone	Completed	No (No)	Yes (Yes)
NCT02363322	Treatment (1/2)	ZMapp	A multicenter randomized safety and efficacy study of putative investigational therapeutics in the treatment of patients with known Ebola infection [77]	Guinea, Liberia, Sierra Leone	Ongoing, not recruiting	No (No)	Yes (Yes)
NCT02333578	Treatment (NS)	Convalescent plasma	A phase I/II pilot clinical trial to evaluate the efficacy and safety of Ebola virus disease (EVD) convalescent plasma (ECP) for treatment of EVD	Liberia	Recruiting	No (No)	Yes (Yes)
NCT02342171	Treatment (2/3)	Convalescent plasma	Emergency evaluation of convalescent plasma for ebola viral Disease (EVD) in Guinea [39] [30] [78]	Guinea (MSF ETC Conakry)	Completed	Yes (Yes)	No
ISRCTN13990511	Treatment (2/3)	Convalescent Plasma	Convalescent plasma for early Ebola virus disease in Sierra Leone: an open-label, non-randomized, controlled clinical trial	Sierra Leone	Completed	No (No)	No
NCT02509494, PACTR201506001147964	Vaccine (3)	Ad26.ZEBOV MVA-BN-Filo	A staged Phase 3 study, including a double-blinded controlled stage to evaluate the safety and immunogenicity of Ad26.ZEBOV and MVA-BN-Filo as candidate prophylactic vaccines for Ebola	Sierra Leone	Recruiting	No (No)	Yes (Yes)
NCT02575456	Vaccine (2)	Ad5-EBOV	A single-center, randomized, blind, Phase II clinical trial to evaluate the safety and immunogenicity of the Adenovirus Type 5 Vector Based Ebola Virus Disease Vaccine (Ad5-EBOV) in healthy adults in Sierra Leone	Sierra Leone	Completed	No (No)	Yes (Yes)
NCT02876328	Vaccine (2)	Ad26.ZEBOV, rVSVΔG/ ZEBOV-GP MVA-BN-Filo	Partnership for research on Ebola vaccinations (PREVAC)	Guinea, Liberia	Recruiting	No (No)	Yes (Yes)
PACTR201503001057193	Vaccine (3)	rVSVΔG/ ZEBOV-GP	A randomized trial to evaluate Ebola vaccine efficacy and safety in Guinea, West Africa. Part A: A randomized trial of ring vaccination to evaluate Ebola vaccine efficacy and safety in Guinea, West Africa. Part B: Safety and immunogenicity of rVSVΔG/ ZEBOV-GP among frontline workers [43] [72]	Guinea, Sierra Leone	Completed	Yes (Yes)	Yes (self-reported, non-obligatory test offered)
NCT02378753, PACTR201502001037220	Vaccine (2/3)	rVSVΔG/Z EBOV-GP	rVSVΔG-ZEBOV Ebola prevention vaccine evaluation in Sierra Leone (STRIVE)	Sierra Leone	Completed	No (No)	Yes (Yes)
NCT02344407	Vaccine (2)	rVSVΔG/ ZEBOV-GP, ChAd3-EBOZ	Partnership for research on Ebola vaccines in Liberia (PREVAIL)	Liberia	Active, not recruiting	No (No)	Yes (Yes)

Source: WHO International Trial Registry Platform (http://apps.who.int/trialsearch/Default.aspx) and referenced publications

^a^ *NS* not specified, ^b^test-negative pregnancy test required

### WHO ethics review committee considerations

The WHO-ERC reviewed all protocols for studies supported or sponsored by WHO, four protocols at the request of the Médecins Sans Frontières Ethics Review Board and one for the Rapid Assessment of Potential Interventions & Drugs for Ebola (RAPIDE) Consortium [16, 31, 32]. The WHO-ERC applied the Council for International Organizations of Medical Sciences guidelines [33] and followed the recommendations of a WHO panel of external experts convened to provide ethical guidance on use of unregistered interventions for treatment or prevention of EBOV in a context in which patients were managed with no, or limited, clinical trial data [34]. The WHO panel counselled use of unregistered interventions in the epidemic, conditional upon evidence from laboratory and animal studies. The panel also emphasized that in prioritizing and allocating interventions “children and pregnant women should be considered particularly vulnerable [because of their higher mortality rates]... and given special protection when receiving such interventions” [34]. In the face of the long history of exclusion of pregnant women from clinical trials [9, 15, 35–37], this recommendation was remarkable and important; the WHO-ERC understood that these groups were to be provided preferential access to interventions. For the WHO-ERC, the virtual certainty of fetal/neonatal loss invalidated exclusion of pregnant women because of risk to the fetus; the high maternal mortality in past Ebola outbreaks favored their inclusion for clinical and ethical reasons. Other ethical considerations dictated that pregnant women should be accorded the same autonomy as non-pregnant adults: pregnant women had a greater interest in and right to decide about their own and their fetus’ health than sponsors, researchers, regulators or ethics committees. The WHO-ERC considered these points equally applicable to vaccine trials in EVD-affected countries that would enroll uninfected participants based on data from Phase I safety and immunogenicity trials and noted that other ethics committees took the same view [31].

By the end of the EBOV epidemic, the WHO-ERC had reviewed 14 protocols for interventional trials as well as two MEURI protocols [16]. These included studies of brincidofovir [32] and favipiravir [38], a study with convalescent plasma [39] and several phases of the rVSVΔG/ZEBOV-GP vaccine [40–43] and the ChAd3-EBO-Z vaccine [44, 45]. All vaccine protocols, including those in affected countries, excluded pregnant women. The brincidofovir trial excluded pregnant women on the basis of embryotoxicity in animal studies without comment on the relevance of these data for a disease resulting in 100% human fetal loss; the favipiravir trial could not include pregnant women because the sponsors were unable to get insurance coverage despite strong recommendations for inclusion from the WHO-ERC, MSF Ethics Review Board, and Inserm Institutional Review Board [31]. The WHO-ERC requested investigators of all treatment and vaccine trials in the affected countries to reconsider exclusion of pregnant women based on benefit-risk assessment, but the requested amendments were not submitted. Therefore, the WHO-ERC faced the difficult dilemma of granting approval for immediate trial start (with potential benefit for the many participants the protocols included) or withholding approval until pregnant women were either included or their exclusion justified. The latter choice would delay trial start in the context of an epidemic for which mortality was high and speed of intervention was essential. Since agreement to include pregnant women would require consensus between numerous parties (including sponsors), which would take time and delay trial start substantially (or possibly indefinitely), WHO-ERC did not make inclusion of pregnant women a prerequisite for WHO-ERC clearance [16]. When interim analysis of the efficacy and safety data in non-pregnant adults in the rVSVΔG/ ZEBOV-GP vaccine trial showed benefit, the WHO-ERC (and the Data Safety Monitoring Board) pointed out the high incidence and mortality rates in children and pregnancy and unsuccessfully sought inclusion of the latter, or justification of exclusion. Forty-two pregnant women were denied participation [43]. However, since the trial excluded pregnant women on the basis of self-reported pregnancy status (pregnancy tests were offered, but not obligatory), more than 20 other pregnant women received the vaccine [Henao-Restrepo and MSF, personal communication] [43].

### Data from the 2013–2016 EVD epidemic that will inform the design of studies in future EBOV outbreaks

The 2013–2016 EVD epidemic permitted better estimates of CFRs and factors impacting survival rates and determination of persistence of EBOV in different body fluids.

The CFR across both sexes was 62.9% (95% CI: 61.9–64.0%) declining from 69.8% (95% CI: 58.6–79.2) to around 39% (95% CI: 25.7–54.3%) from July 2015 to September 2015. Survival was highest in those under 5 years (75.6%) and above 75 years (83.8%), a pattern similar in all three countries [2]. Both sexes were equally susceptible to infection [46]. In all countries, time from initial symptoms to hospitalization was approximately 0.5 days shorter for women [46]. CFRs were significantly lower for women: 63% (95% CI: 61.6–64.4, *n* = 4756) versus 67.1% for men (95% CI, 65.8–68.5, *n* = 4637), *p* < 0.001; the survival difference was significant after adjustment for age, clinical symptoms, and intervals between onset and hospitalization [46].

Despite the size of the epidemic, and the opportunity, information on pregnancy and pregnancy outcomes was not systematically obtained. Available data are shown in Table 1B; some analyses are ongoing. The maternal CFR estimated from these data is 55% (44/80) [46–58] excluding approximately 20 pregnant vaccinated women [43]; maternal CFR is not statistically significantly different from the CFR of women overall. All surviving mothers experienced miscarriages or stillbirths [49–51, 57, 58] and two women died with the fetus in utero [47, 48]. The only surviving baby was born to a woman who had received favipiravir under a MEURI protocol and died. Authorization was given to MSF to treat the newborn, but not the mother, with ZMapp [56] [59]. The reasons for high fetal mortality may be related to EBOV placental preference and consequent high viral load in utero, as samples from amniotic fluid, placenta, and fetuses tested positive for EBOV [52]. Live-born babies appear to have been preterm births and preterm babies normally have a high mortality risk. In EBOV-affected countries where babies are often exclusively breastfed immediately after birth (and there may not be a safe alternative to breastfeeding available), the absence of a surviving mother or the inability of an EBOV-infected survivor to breastfeed places a surviving baby at risk of death.

As of 2 February 2016, between 10,000 and 17,000 EBOV survivors were reported compared with 1000 survivors from all previous epidemics combined [2, 60, 61]. Compared to blood used for determining cure, clearance of EBOV is delayed (sometimes for months) in immunologically protected fluids/body compartments including semen [62, 63], ocular tissues [64], breastmilk [65], vaginal secretions [66] and the central nervous system [60, 67]. Mother-to-child transmission of EBOV can occur through body fluids in utero, during delivery, contact after birth, and breastmilk, even when the woman is asymptomatic [48]. Among 70 EBOV survivors who conceived post-recovery, 15/68 miscarried and two survivors elected to terminate their pregnancies; four neonates were stillborn (3/4 conceived within two months of discharge of the Ebola Treatment Unit). While still sparse, the data suggest that pregnancies shortly after recovery also increase the risk for poor outcomes [68].

## Conclusions

In this epidemic, a positive diagnosis meant a high probability of mortality; interventions yet to be proven effective provided the best chance of avoiding death. Despite an 89% maternal CFR and near certain fetal loss in previous outbreaks (i.e. little chance of harming the fetus by administering an experimental intervention), pregnant women were systematically excluded from all drug and vaccine trials. Their automatic disqualification denied pregnant women the potential for benefit given to others. EBOV-infected pregnant women as a class were also harmed because knowledge to protect them (and their fetuses) now lags behind knowledge for other groups. Results from studies that excluded pregnant women cannot be automatically extrapolated to pregnancy. This lack of data specific to pregnancy will negatively impact the health of pregnant women and their access to interventions in the next outbreak.

Each case of EBOV infection during pregnancy in previous outbreaks has resulted in the death of the woman or her fetus; no mother-baby pair has ever survived. Therefore, EBOV infection satisfied two conditions that should have driven inclusion of pregnant women in trials: firstly, EBOV is a life-threatening infection and chance of survival constitutes an important medical benefit. Secondly, with 100% fetal/neonatal death without intervention, investigational treatment of the mother could not place the fetus at “greater than minimal” added risk. Importantly, by excluding pregnant women, sponsors, investigators, insurance companies, and others influencing protocol provisions violated the autonomy of pregnant women and their right to decide on research participation for themselves, a fundamental ethical principle.

The largest ever EVD epidemic provided ideal conditions to deviate from usual practice for the immediate potential benefit of EBOV-infected pregnant women and the potential benefit of pregnant women in future outbreaks or epidemics. This opportunity was lost. It is time to stop “protecting” pregnant women by excluding them from trials without their consent, and time to insist on rigorous justification of exclusion, thus according pregnant women the same rights and opportunities we offer other adults.

## Abbreviations

EBOV: Ebola virus
EVD: Ebola virus disease
MEURI: Monitored Emergency Use of Unregistered and Experimental Interventions
WHO: World Health Organization
WHO-ERC: World Health Organization Ethics Review Committee
